# The role of adjuvant chemotherapy after radical surgery in patients with lymph node-positive bladder cancer or locally advanced (pT3, pT4a) bladder cancer: a meta-analysis and systematic review

**DOI:** 10.1097/JS9.0000000000001954

**Published:** 2024-07-17

**Authors:** CaiXia Chen, ChongJian Wang, HaoTian Huang, HongYuan Li, Zhi Wen, Yang Liu, Xue-song Yang

**Affiliations:** Department of Urology, Affiliated Hospital of North Sichuan Medical College, Nanchong, People’s Republic of China

**Keywords:** bladder cancer, chemotherapy, meta-analysis, radical cystectomy

## Abstract

**Purpose:**

This study aims to evaluate the impact of adjuvant chemotherapy (AC) on survival outcomes in patients with lymph node-positive bladder cancer or locally advanced (pT3, pT4a) bladder cancer after surgery. The authors also seek to identify which patients with pN+ bladder cancer are most likely to benefit from AC after radical cystectomy (RC).

**Methods:**

The authors searched databases including Embase, PubMed, Cochrane, and ClinicalTrials.gov to identify relevant literature published in English up to February 2024. The authors used Stata to compare various parameters. The study has been registered in PROSPERO.

**Results:**

A total of 21 studies were analyzed, including 1 randomized controlled trial, 6 prospective studies, and 14 retrospective studies, encompassing 12 888 patients. The meta-analysis showed that for patients with lymph node-positive bladder cancer, the AC group had higher overall survival (OS) [*I* ^2^=58.2%, hazard ratio (HR) 0.69; 95% CI: 0.57–0.83; *P*=0.019] and recurrence-free survival (*I* ^2^=66.6%, HR 0.71; 95% CI: 0.57–0.89; *P*=0.006) compared to the RC group. For patients with pT3 and pT4a bladder cancer, the AC group had higher OS (*I* ^2^=57.3%, HR 0.77; 95% CI: 0.67–0.89; *P*=0.022) and cancer-specific survival (*I* ^2^=47.2%, HR 0.75; 95% CI: 0.64–0.88; *P*=0.0048) compared to the RC group. At the same time, according to the different chemotherapy regimens, the authors divided the cisplatin-based chemotherapy regimen and carboplatin-based chemotherapy or other regimens into two subgroups for analysis, and found that the OS (*I* ^2^=41.4%, HR 0.64; 95% CI: 0.51–0.80; *P*=0.000) was better than carboplatin and other chemotherapy regimens (*I* ^2^=64.1%, HR 0.77; 95% CI: 069–0.86; *P*=0.000); lymph node density was found to be an independent predictor of OS (HR=1.6; 95% CI: 1.31–1.95; *P*=0.0000).

**Conclusion:**

This study found that postoperative AC improves OS, cancer-specific survival, and recurrence-free survival in patients with pT3, pT4a. It was also confirmed that cisplatin-based chemotherapy regimen was more beneficial for patients with bladder cancer; and lymph node-positive bladder cancer. Additionally, our analysis revealed that patients with lymph node-positive bladder cancer benefit more from postoperative AC. It was further demonstrated that cisplatin-based chemotherapy regimens are more beneficial than other regimens for patients with locally advanced bladder cancer.

## Introduction

Highlights

- The meta-analysis showed that for patients with lymph node-positive bladder cancer, the adjuvant chemotherapy group had higher overall survival (OS) [*I* ^2^=58.2%, hazard ratio (HR) 0.69; 95% CI: 0.57–0.83; *P*=0.019] and recurrence-free survival (*I* ^2^=66.6%, HR 0.71; 95% CI: 0.57–0.89; *P*=0.006) compared to the radical cystectomy group.
- For patients with pT3 and pT4a bladder cancer, the adjuvant chemotherapy group had higher OS (*I* ^2^=57.3%, HR 0.77; 95% CI: 0.67–0.89; *P*=0.022) and cancer-specific survival (*I* ^2^=47.2%, HR 0.75; 95% CI: 0.64–0.88; *P*=0.0048) compared to the radical cystectomy group.
- Lymph node density was found to be an independent predictor of OS (HR=1.6; 95% CI: 1.31–1.95; *P*=0.0000).

Globally, bladder cancer ranks as the 10th most common malignancy, being the 6th most common in men and ranking below 10th in women, with the 12th highest mortality rate among malignancies^1^. In Europe and North America, bladder cancer is the 4th most common malignancy in men, following prostate, lung, and colorectal cancers, and ranks below 10th in women^2^. In 2020, there were ~573 000 new cases of bladder cancer and 213 000 deaths worldwide. The age-standardized incidence rates were 9.5 per 100 000 for men and 2.4 per 100 000 for women^1^; the age-standardized mortality rates were 3.3 per 100 000 for men and 0.9 per 100 000 for women^1^. There are regional differences in age-standardized incidence and mortality rates, with the highest incidence rates found in Southern Europe, Western Europe, and North America^1^. Mortality rates are higher in developing regions compared to developed regions. The risk of bladder cancer recurrence remains high, with reported 5-year recurrence-free survival (RFS) and overall survival (OS) rates after surgery ranging from 48 to 70% and 57 to 60%^3–5^, respectively. Once patients experience recurrence after radical cystectomy (RC), their median survival time is about 15 months^6^, and even shorter if they do not receive salvage chemotherapy^7^.

Despite consistent level I evidence supporting the benefits of neoadjuvant chemotherapy (NAC), it has not been widely adopted in practice^8^. Instead, postoperative systemic chemotherapy [adjuvant chemotherapy (AC)] has been used more frequently. David *et al*.^9^ reported that the utilization rates of NAC and AC in stage III bladder cancer were 1.4 and 10.4%, respectively. A recent meta-analysis of nine randomized controlled trials (RCTs), including 945 participants, demonstrated a statistically significant benefit of AC on OS in muscle-invasive bladder cancer [hazard ratio (HR), 0.77; 95% CI: 0.59–0.99; *P*=0.049]^10^.

However, the efficacy of postoperative AC in patients with locally advanced (pT3/T4) and/or lymph node-positive (pN+) bladder cancer remains controversial. It is also unclear whether the efficacy of AC in lymph node-positive (pN+) bladder cancer is associated with clinical and pathological characteristics. Approximately 20–25% of patients with muscle-invasive bladder cancer are found to have pN+ disease after undergoing RC and lymph node dissection^10,11^.

Therefore, this study aims to evaluate the impact of postoperative AC on survival outcomes in patients with locally advanced (pT3/T4) and/or pelvic lymph node-positive (pN+) bladder cancer. We also seek to identify which pN+ bladder cancer patients are most likely to benefit from AC after RC, providing clinicians with the latest evidence for clinical decision-making.

## Methods

### Materials and methods

We followed the guidelines of the Preferred Reporting Items for Systematic Reviews and Meta-Analyses (PRISMA)^12^, Cochrane Handbook for Systematic Reviews of Interventions, and A MeaSurement Tool to Assess systematic Reviews 2 (AMSTAR2)^13^ to conduct the systematic review and meta-analysis; and it is registered in PROSPERO.

### Literature search strategy

We conducted a comprehensive search on Embase, PubMed, Cochrane, and ClinicalTrials.gov for relevant literature up to February 2024. We used the following MeSH terms and keywords for searching: ‘Chemotherapy, Adjuvant’ and ‘Radical Cystectomy’ and ‘node-positive bladder cancer’. Additionally, we manually searched and reviewed relevant literature to avoid any omissions, and the references included only studies reported in English.

### Inclusion and exclusion criteria

Using the PICOS (Patient, Intervention, Comparison, Outcome, Study Type) method to establish inclusion criteria: P) Patients with pathologically diagnosed pelvic lymph node metastasis or locally advanced bladder cancer after RC; I) Experimental group patients receive systemic chemotherapy (any regimen); C) Control group patients do not receive systemic chemotherapy; O) One or more of the following outcomes: OS, disease-free survival, tumor-specific survival; S) Cohort studies, prospective studies, or RCTs. Exclusion criteria include: (1) Noncomparative studies; (2) Editorial comments, conference abstracts, case reports, unpublished studies, or commentaries; (3) Studies for which analysis data cannot be obtained.

### Study screening and selection

Data extraction and risk assessment were independently conducted by two researchers. Initially, the titles and abstracts of the retrieved articles were reviewed, and full-text evaluation was performed for articles meeting the inclusion criteria. Data extraction and analysis were carried out by two independent researchers, and any discrepancies were resolved through consultation with a third researcher.

### Data items

We extracted the following data from the articles meeting the inclusion criteria: type of included studies (RCT or other types), total number of patients, age, sex, Pathologic T stage, Pathologic N stage, OS, disease-free survival, tumor-specific survival, etc. Any discrepancies were resolved through consensus or consultation with a third researcher.

### Bias risk assessment

We used the Cochrane bias risk assessment tool to evaluate potential biases in RCTs. This tool examines five domains of RCTs: the randomization process (selection bias), deviations from intended interventions (performance bias), incomplete outcome data (attrition bias), measurement of the outcome (detection bias), and selection of the reported result (reporting bias). These are categorized as low risk, unclear risk, and high risk^14^. For non-RCT studies, we utilized the Newcastle–Ottawa Scale (NOS) to assess bias risk. The literature quality was assessed using a semi-quantitative star system, with a total of eight stars, and studies scoring six stars or more were included.

### Statistical analysis

In this study, we conducted statistical analyses using Stata V17 software. Results are reported as 95% CIs and risk ratios (RR) for binary variables, and as mean differences (MD) for continuous variables. For studies that only provided median, quartiles, or ranges, data were transformed into mean (MD) and SD^15,16^. We used the *I* ^2^ statistic to assess heterogeneity among studies, with *I* ^2^<50% indicating mild heterogeneity, 40–60% indicating moderate heterogeneity, 60–90% indicating significant heterogeneity, and 90–100% representing substantial heterogeneity^17^. The *I* ^2^ statistic and Cochran’s *Q* test were used to calculate the heterogeneity of the studies^17^. A *P*-value <0.05 was considered statistically significant.

### Sensitivity analysis

To ensure the stability of our results, we conducted sensitivity analysis using Stata software and employed the leave-one-out method to detect heterogeneity among included studies. This method sequentially excludes each study from the pooled effect. Additionally, we examined the robustness by considering the size of cohort studies, which could be a source of heterogeneity. However, it should be noted that sensitivity analysis cannot be performed when comparing three or fewer studies.

### Publication bias

**Figure 1:**
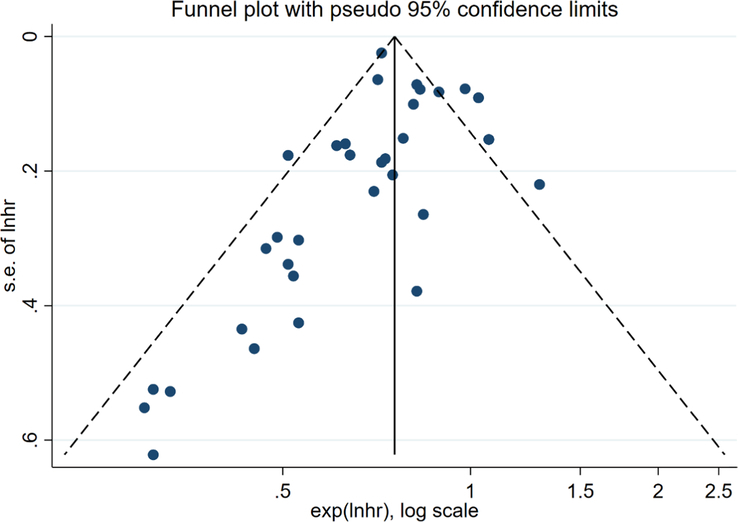
Funnel plot of anastomtic leak.

**Figure 2:**
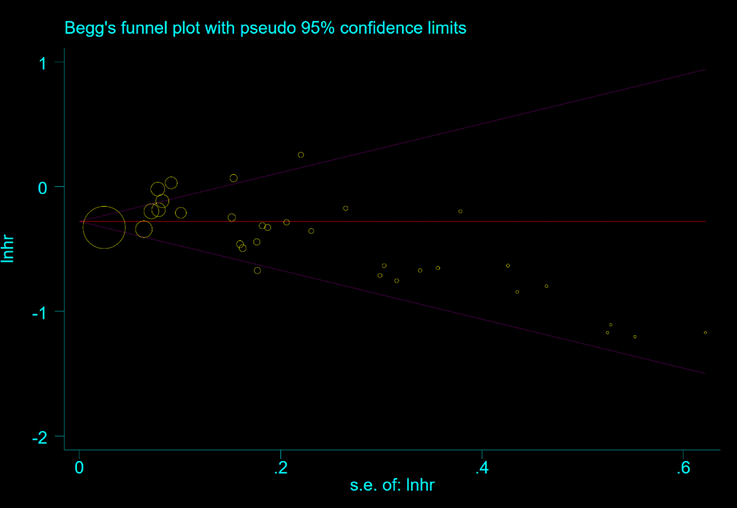
Funnel plot of Begg.

Funnel plots were used to assess potential publication bias in the meta-analysis. As shown in Figure 1, the two sides of the funnel plot were not completely symmetric with naked eyes, so we conducted the Begg test as shown in Figure 2 [*P*=0.212 (*P*>0.05 indicates low publication bias)], and the risk of publication bias in this study was low.

## Result

### Baseline characteristics

**Figure 3:**
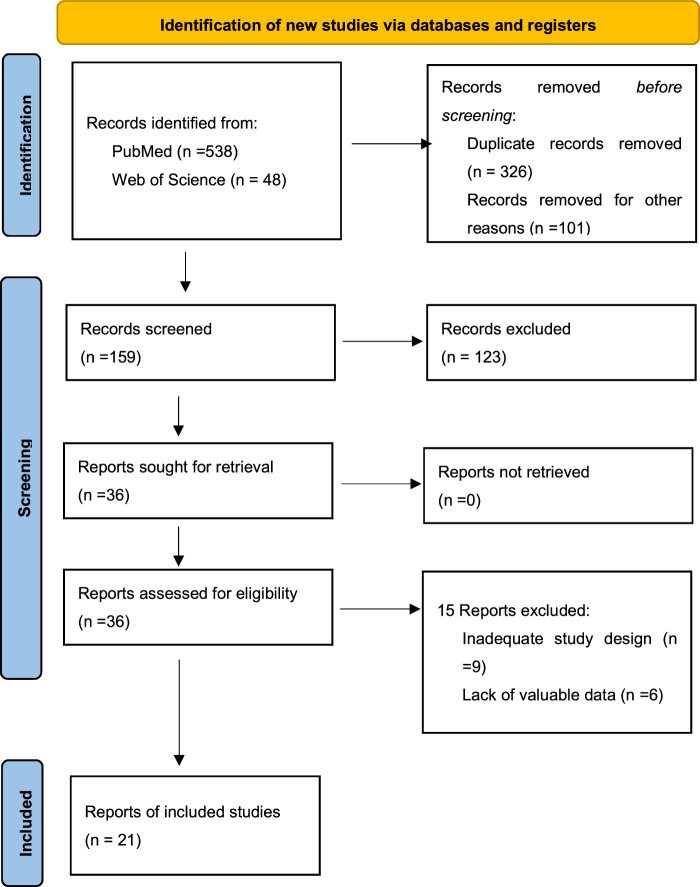
RPISMA Flowchart of the study selection process.

Figure 3 shows that initially, we identified 586 articles, of which 36 remained after removing duplicates and ineligible articles. After reviewing titles and abstracts, eight studies were excluded. Following full-text reading and screening, 7 articles were further excluded, leaving a total of 21 articles for inclusion in this meta-analysis, comprising 12 888 patients. These 21 studies were published between 1994 and 2022 (Table 1). Among them, there was 1 randomized controlled trial, 6 prospective studies, and 14 retrospective studies, with follow-up periods ranging from 5 to 10 years. All included bladder cancer patients underwent RC and pelvic lymph node dissection. The male-to-female ratio was significantly higher among the included patients. The majority of patients in the included studies had a Pathologic T stage of ≥pT3 and lymph node-positive Pathological N stage. Baseline data for all studies are summarized in Table 2.

**Table 1:** Demographic characteristics of the include studies.

Study	Design	Size		Age (years)		Sex (*n*)				Pathologic T Stage				Pathological N Stage				AC
		1	2	1	2	1		2		1		2		1		2		
						Male	Female	Male	Female	<pT3	≥pT3	<pT3	≥pT3	pN0	pN+	pN0	pN+	
Afferi2022	Retrospective	281	281	64.02±2.28	64.91±2.28	239	42	238	43	74	207	74	207	0	281	0	281	Carboplatin-based
Luca2022	Retrospective	183	61	68.00±2.95	69.08±2.37	0	34	54	7	143	40	47	14	0	183	0	61	Carboplatin-based
Sahyun2019	Retrospective	122	159	66.3	61.2	102	20	142	17	13	109	14	145	0	122	0	159	GC (84.3%), MVAC (15.7%)
Malte2018	Prospective	140	84	70.75±9.56	64.58±8.74	106	34	74	10	10	130	13	71	80	60	22	62	NA
Matthew2016	Retrospective	4360	1293	69.63±9.91	63.70±10.07	3205	1155	957	336	0	2367	0	369	609	1384	102	822	NA
Lucca20152L	Retrospective	649	874	69.96±2.25	64.97±2.19	496	153	666	208	168	481	223	651	0	649	0	874	MVAC, MVEC, GC
Kanatani2015K	Retrospective	22	39	71.71±4.18	62.30±2.56	21	1	35	5	3	19	5	34	12	10	18	21	MVAC/GC
Tabata2015	Retrospective	34	31	66.11±3.58	66±2.43	28	8	28	4	0	34	0	31	36	8	20	21	MVAC(81%)/GC (3%)/ other regimens (16%)
Zehnder2014	Retrospective	251	270	69.61±9.07	63.83±9.18	186	65	209	61	56	195	78	192	0	251	0	270	NA
Marisa2014	Retrospective	NA	NA	NA	NA	NA	NA	NA	NA	NA	NA	NA	NA	NA	NA	NA	NA	NA
Daniel2014	Retrospective	80	595	63±2.48	70.01±2.11	66	14	461	133	23	57	235	360	12	68	460	143	NA
Nicholas2012	Retrospective	NA	NA	NA	NA	NA	NA	NA	NA	NA	NA	NA	NA	NA	NA	NA	NA	NA
Cognetti2011	RCT	86	97	62.07±7.98	63.14±7.44	75	11	90	7	23	57	235	360	12	88	452	143	NA
Berkan2010	Retrospective	46	32	60.29±10.17	60.04±9.43	29	3	14	3	20	66	32	65	49	37	47	50	NA
STUDER1994	Prospective	40	37	59.39±5.56	63.89±4.46	27	13	30	7	NA	NA	NA	NA	NA	4	NA	3	Clsplatin
SUZUKI2004	Retrospective	15	16	66.48±10.	57.17±10.46	13	2	14	2	3	12	2	14	0	15	0	16	M-VAC/MEC
lehmann2006	Prospective	23	26	62.7	58.8	19	4	22	4	6	17	3	23	10	13	10	16	MVAC/ MVEC
Jinsung2007	Prospective	42	43	NA	NA	38	4	39	4	9	33	7	36	0	42	0	43	MVAC/GC
Osawa2009	Retrospective	35	25	NA	NA	NA	NA	NA	NA	NA	NA	NA	NA	NA	NA	NA	NA	NA
Alexander 2020	Prospective	47	53	60.58±10.71	61±9.14	41	6	49	4	NA	NA	NA	NA	26	21	27	26	GC (84.3%), MVAC (15.7%)
Bharadwaj2022	Prospective	1471	945	64.65±8.16	63±8.91	1149	322	720	225	137	1334	170	776	934	537	305	640	NA

GC, gemcitabine and cisplatin, MEC, methotrexate, epirubicin and cisplatin, MVAC, methotrexate, vinblastine, adriamycin and cisplatin.

**Table 2:** The demographics of the studies.

Variable	Number of studies with available data	WMD/RR	95% CI	*P*
Age (years)	15	1.748	−0.706–4.202	0.179

OR, odds ratio; WMD, weighted mean difference.

### Quality assessment

**Figure 4:**
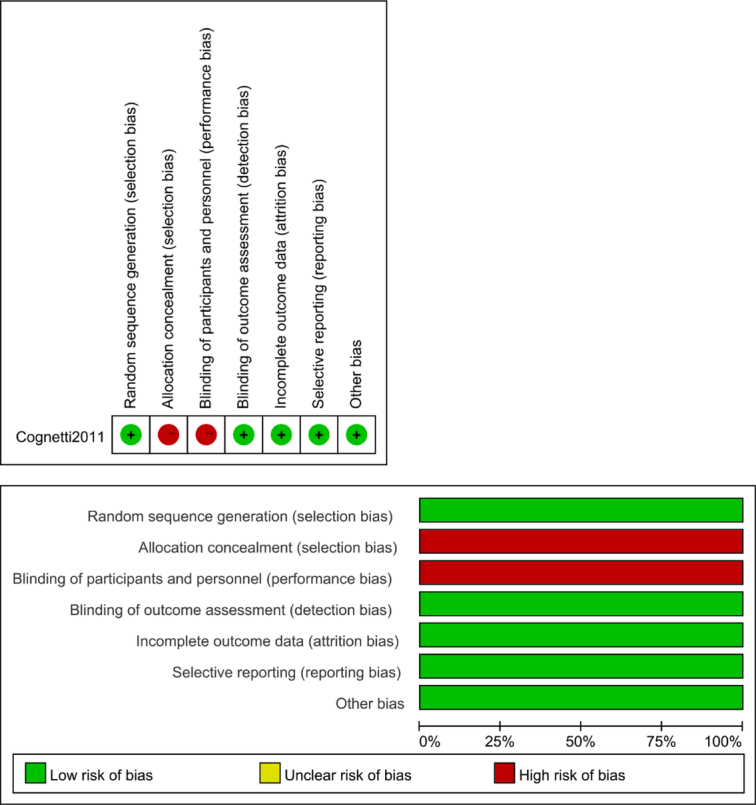
Quality assessment of randomized controlled studies.

A score of six stars or higher on the NOS is defined as high-quality literature. All of the 6 prospective studies and 14 retrospective studies included in the analysis scored 6 stars or higher on the NOS, indicating good quality. Cognetti’s study had a high risk of bias due to the lack of blinding of participants and personnel (performance bias) and blinding of outcome assessment (detection bias)^18^. Quality assessment of RCTs is detailed in Figure 4, and for non-RCTs, please refer to Table 3.

**Table 3:** Study quality of case–control studies based on the Newcastle–Ottawa scale.

NOS		Selection				Comparability	Exposure			Total Score
		1	2	3	4	5	6	7	8	
Afferi2022	retrospective	★	★	★	★	★	★	★	★	8/High
Luca2022	retrospective	★	★	★	★	★		★	★	7/High
Sahyun2019	retrospective	★	★	★	★	★	★	★	★	7/High
Malte2018	prospective	★	★	★	★	★	★	★	★	7/High
Matthew2016	retrospective	★	★		★	★	★		★	6/High
Lucca20152L	retrospective	★	★	★	★	★	★	★	★	8/High
Kanatani2015K	retrospective	★	★	★	★	★	★		★	7/High
Tabata2015	retrospective	★	★	★	★	★		★	★	7/High
Zehnder2014	retrospective	★	★	★	★	★	★		★	7/High
Marisa2014	retrospective	★	★	★	★	★	★	★	★	8/High
Daniel2014	retrospective	★	★	★	★	★	★	★		7/High
Nicholas2012	retrospective	★	★	★	★	★	★		★	7/High
Berkan2010	retrospective	★	★	★	★	★		★	★	7/High
STUDER1994	prospective	★	★		★	★	★		★	6/High
SUZUKI2004	retrospective	★	★		★	★	★		★	6/High
lehmann2006	prospective	★	★		★	★	★		★	6/High
Jinsung2007	prospective	★	★	★	★	★	★		★	7/High
Osawa2009	retrospective	★	★		★	★	★		★	6/High
Alexander 2020	prospective	★	★	★	★	★	★	★	★	8/High
Bharadwaj2022	prospective	★	★		★	★	★		★	6/High

1:Representativeness of the exposed cohort; 2: Selection of the nonexposed cohort; 3: Assessment of exposure; 4:Demonstration that outcome of interest was not present at the start of the study; 5:Comparability of cohorts based on the design or analysis; 6: Ascertainment of outcome; 7:Long enough follow-up for outcomes to occur; 8: Adequacy of follow-up of cohorts.

### Outcome analysis

#### Lymph node-positive bladder cancer OS

**Figure 5:**
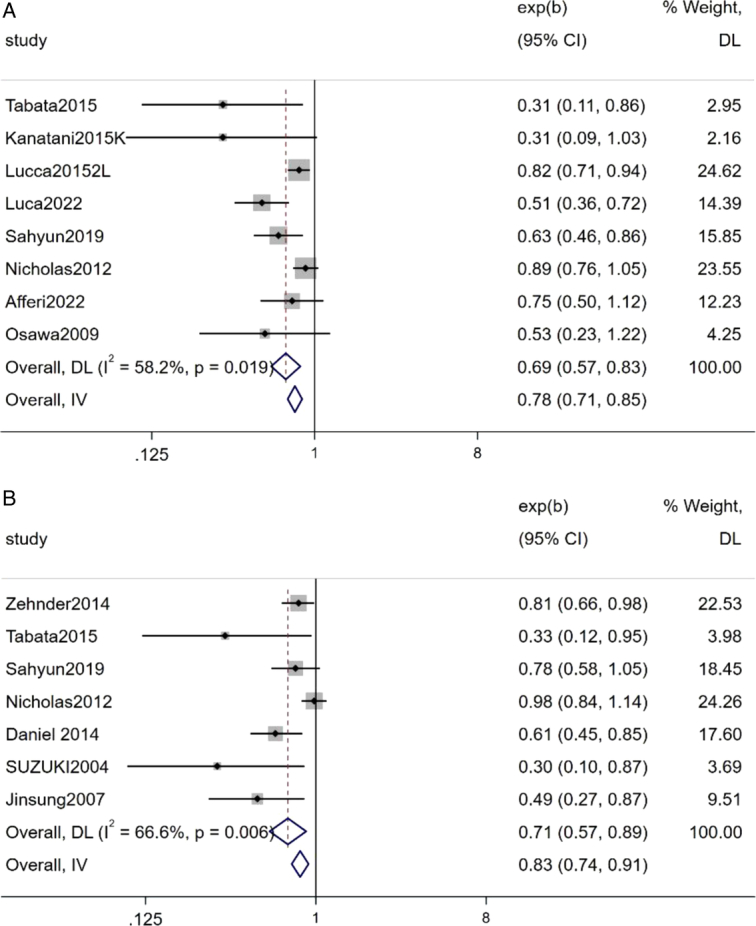
Forest plot A: lymph node-positive bladder cancer OS; B: lymph node-positive bladder cancer RFS.

Summary results from eight studies revealed that bladder cancer patients undergoing RC with pelvic lymph node dissection, lymph node-positive patients who did not receive AC had a 69% OS compared to patients receiving chemotherapy, and this difference was statistically significant (Z=3.87, *P*<0.05) (Fig. 5).

#### Lymph node-positive bladder cancer RFS

Summary results from five studies revealed that bladder cancer patients undergoing RC with pelvic lymph node dissection, lymph node-positive patients who did not receive AC had a 71% RFS compared to patients receiving chemotherapy, and this difference was statistically significant (Z=2.99, *P*=0.003) (Fig. 5).

#### Cancer-specific survival (CSS)

**Figure 6:**
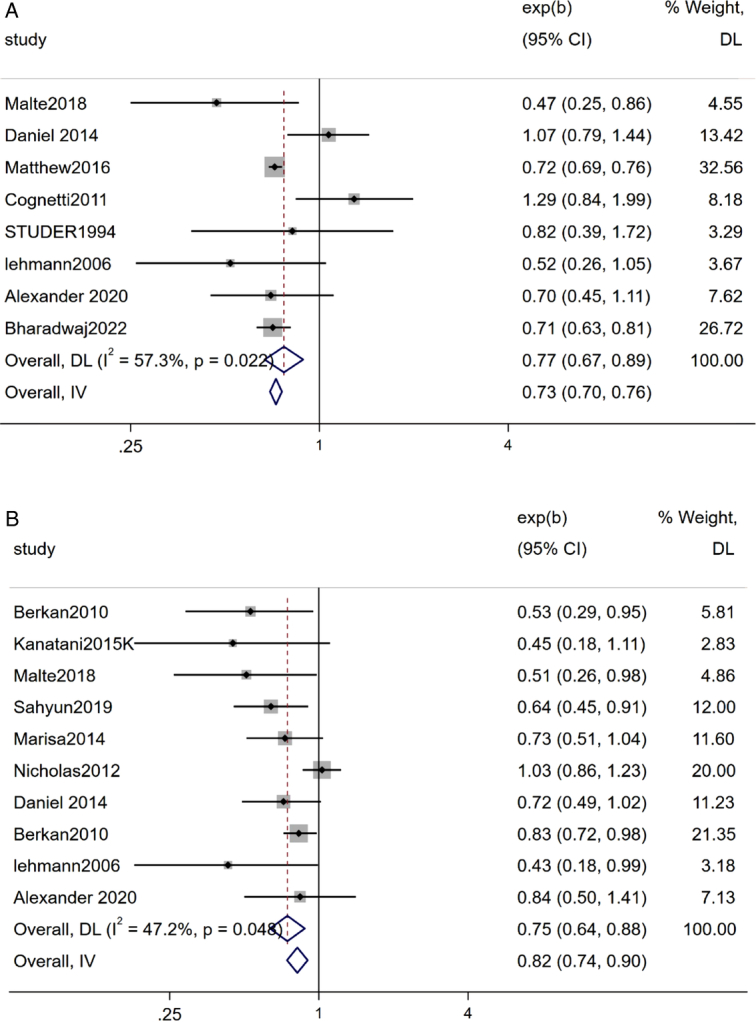
Forest plot A: OS; B: cancer-specific survival (CSS).

Summary results from 10 studies revealed that bladder cancer patients undergoing RC with pelvic lymph node dissection, patients who did not receive AC had a tumor-specific survival of 75% compared to patients receiving chemotherapy, and this difference was statistically significant (Z=3.54, *P*<0.05). (Fig. 6).

### Overall OS

Summary results from eight studies revealed that bladder cancer patients undergoing RC with pelvic lymph node dissection, patients who did not receive AC had a total survival of 77% compared to patients receiving chemotherapy, and this difference was statistically significant (Z=3.61, *P*<0.05) (Fig. 6).

#### OS (Cisplatin-based chemotherapy regimen)

**Figure 7:**
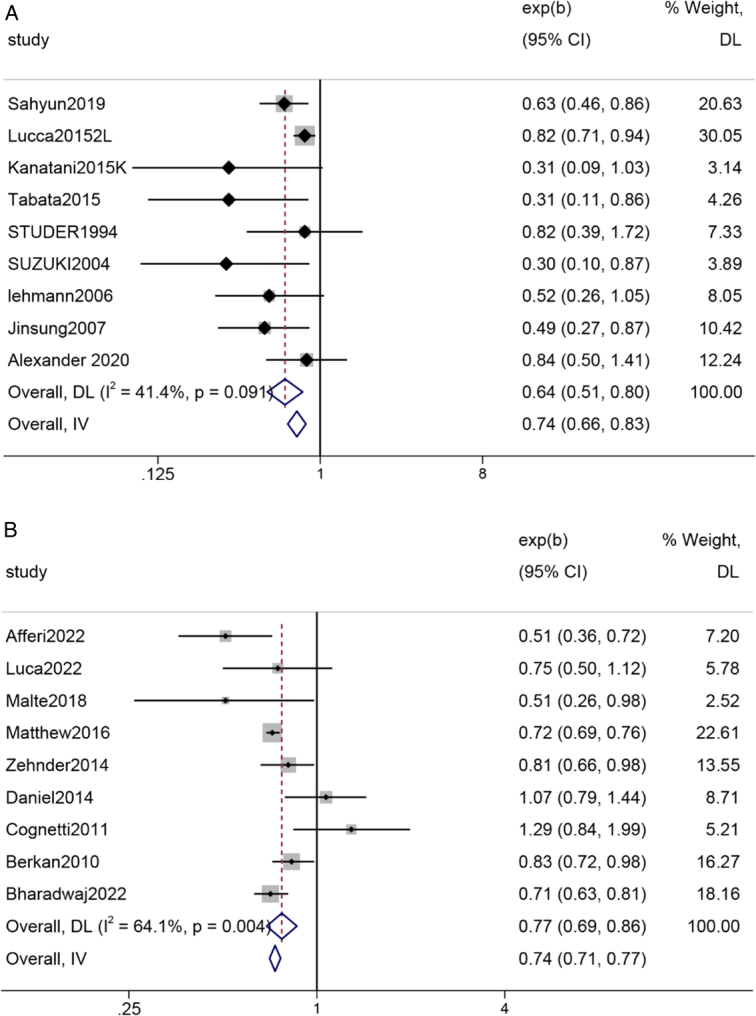
Forest plot A: OS (Chemotherapy regimen with cisplatin); B: OS (Chemotherapy regimens with carboplatin or other regimens).

The pooled results of nine studies showed that for bladder cancer patients who underwent RC and pelvic lymph node dissection, the OS rate of patients who did not receive AC postsurgery was 64% compared to those who received cisplatin-based chemotherapy, and this difference was statistically significant,(Z=3.90, *P*=0.000) (Fig. 7).

#### OS (Carboplatin-based chemotherapy regimen or other regimens)

The pooled results of nine studies showed that for bladder cancer patients who underwent RC and pelvic lymph node dissection, the OS rate of patients who did not receive AC postsurgery was 77% compared to those who received carboplatin-based or other chemotherapy regimens, and this difference was statistically significant (Z=4.539, *P*=0.000) (Fig. 7).

#### Multivariate Cox regression analysis (age)

Summary results from eight studies revealed that in a multivariate Cox regression model, age (≥65 years) was associated with a 1.63-fold increase in the deterioration of OS, and this association was statistically significant (Z=2.319, *P*=0.054) (Fig. 6).

#### Multivariate cox regression analysis (No. of positive nodes)

**Figure 8:**
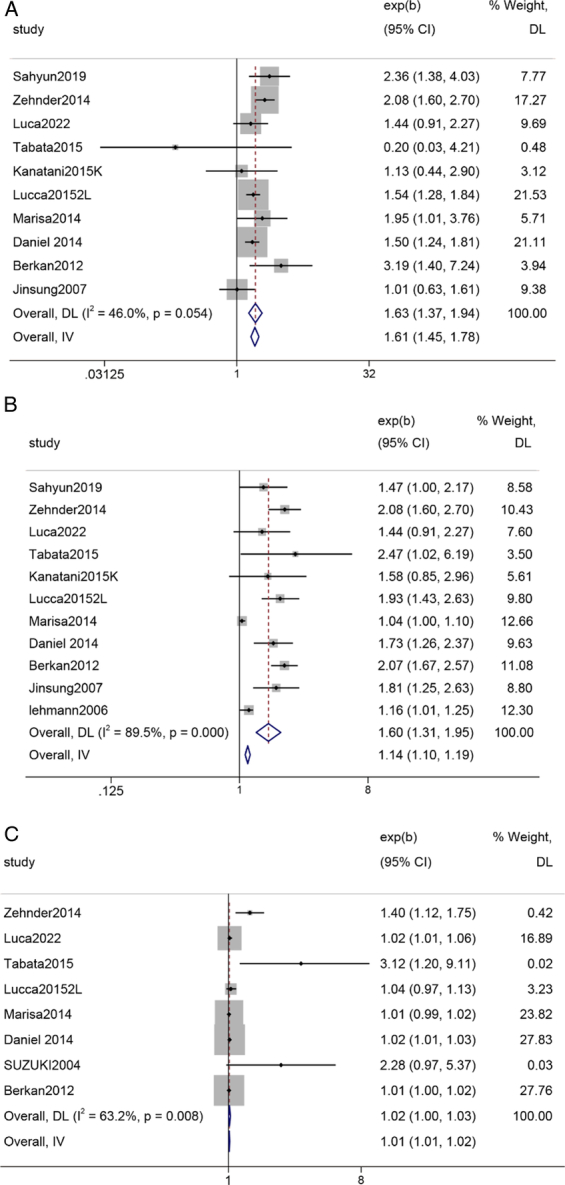
Forest plot A: Multivariate Cox regression analysis (age); B: Multivariate Cox regression analysis (No. of positive nodes) C: Multivariate Cox regression analysis (≥T3).

Summary results from 11 studies revealed that in a multivariate Cox regression model, an increase in lymph node positivity predicted a 1.6-fold deterioration in OS, and this association was statistically significant (Z=4.634, *P*<0.000) (Fig. 8).

#### Multivariate cox regression analysis (≥T3)

Summary results from 10 studies revealed that in a multivariate Cox regression model, patients with pathological staging ≥pT3 predicted a 1.63-fold deterioration in OS, and this association was statistically significant (Z=5.427, *P*=0.008) (Fig. 6).

### Sensibility analysis

Most results showed moderate to low heterogeneity, but greater heterogeneity (66.6%) was observed in lymph node-positive bladder cancer RFS. Sensitivity analysis revealed that the heterogeneity mainly stemmed from the studies by Nicholas 2012 and Zehnder 2014. The reason for the heterogeneity in these studies was that both used chemotherapy regimens other than cisplatin-based regimens, while the other five studies all used cisplatin-based chemotherapy regimens. Moderate heterogeneity (57.3%) was observed in the OS of patients with lymph node-positive pT3 or pT4, and sensitivity analysis identified Matthew 2016 as the primary source of heterogeneity. The reason for the heterogeneity in Matthew 2016 was that the included patients were significantly older, which meant more comorbidities and poorer tolerance to surgery and chemotherapy, leading to lower survival rates.

In the Multivariate Cox regression analysis (No. of positive nodes) group, substantial heterogeneity (89.5%) was observed. Sensitivity analysis identified Sahyun 2019 and Daniel 2014 as the main sources of heterogeneity. The reason for the heterogeneity in Daniel 2014 was the significantly older age of the included patients. For Sahyun 2019, the heterogeneity was due to the older age of patients in the RC group and the higher rate of NAC before RC. Therefore, we must cautiously interpret these results and more studies are needed to confirm this. However, the low or moderate heterogeneity of these results could be misleading because *I* ^2^ can be highly biased in a small number of studies^19^.

## Discussion

Currently, the standard neoadjuvant treatment for locally advanced bladder cancer patients is platinum-based combination chemotherapy. Although NAC can provide chemotherapy early and help determine chemotherapy sensitivity within the body, there is a drawback in delaying treatment for chemotherapy non-responsive patients^18,20^. Therefore, postoperative chemotherapy remains the choice of treatment for most clinicians. Patients with positive lymph nodes (PN+) are usually grouped together with pT3/4 patients, and so far, there has been no specific subgroup analysis for PN+ bladder cancer patients in meta-analyses^21^. Studies have shown a trend indicating that PN+ patients may benefit more from postoperative chemotherapy^14,22^. Some important findings in our study regarding lymph node-positive OS, RFS results, and multivariate Cox regression analysis of OS rate-related variables are worthy of further exploration.

### Lymph node-positive OS and RFS results

Eight articles reported the impact of postoperative AC on OS in the subgroup of lymph node-positive patients. Meta-analysis revealed that among lymph node-positive patients, the prognosis of OS without postoperative AC was 69% compared to patients receiving chemotherapy, and this difference was statistically significant. Seven articles reported the effect of postoperative AC on RFS in the lymph node-positive subgroup. Meta-analysis showed that among lymph node-positive patients, the prognosis of RFS without postoperative AC was 71% compared to patients receiving chemotherapy, and this difference was statistically significant. Moderate heterogeneity was observed among lymph node-positive patients, and sensitivity analysis indicated that the heterogeneity mainly stemmed from the study by Lucca *et al*.^21^, where bladder cancer patients included were older.

The latest European Association of Urology guidelines in 2023 strongly recommend providing platinum-based adjuvant combination chemotherapy for lymph node-positive patients^20^. The definition of RFS is the time interval from the start of surgery to the occurrence of disease recurrence or death due to disease progression. Currently, lymph node metastasis is associated with higher rates of postoperative progression and lower survival rates^23^. Therefore, identifying which patients should undergo postoperative AC more actively is an important step in reducing postoperative metastasis rates and improving disease-free survival rates for patients.

We found that the prognosis of OS in lymph node-positive patients is superior to that of the overall patient population. The study by Luca and colleagues^21^ indicated that only patients with poor prognosis (pTany, PLN≥3) showed significant improvement in OS after receiving adjuvant cisplatin-based chemotherapy (41 vs. 13%; *P*<0.0001), while there was no significant OS improvement observed in the intermediate prognosis group (≥pT3, PLN≤2) and the favorable prognosis group (≤pT2, PLN≤2) after adjuvant cisplatin-based chemotherapy. In the study by Sahyun *et al*.^23^, patients were categorized based on lymph node density into <9%, 9–25%, and ≥25% groups. Among patients with lymph node metastasis <9%, the 3-year OS rates were similar between the AC group and the RC group (58.7 vs. 51.7%, *P*=0.878). However, in patients with 9–25% lymph node metastasis (53.4 vs. 23.7%, *P*=0.003) and ≥25% lymph node metastasis (27.4 vs. 16.1%, *P*=0.032), the AC group was significantly higher than the RC group. These results support the theory that survival outcomes in PN+ bladder cancer population may exhibit heterogeneity^20,24^, and emphasize the prognostic value of PT staging for bladder cancer patients, including in the PN+ subgroup. As previously suggested, the OS prognosis of bladder cancer patients with local organ metastasis is better than that of patients with lymph node metastasis^10,25,26^. Due to the current variations in the definition of lymph node metastasis quantity across studies, meta-analysis is not feasible, necessitating further randomized clinical trials from multiple centers to confirm and validate these findings.

### Multivariable Cox regression analysis of overall survival-related variables in lymph node-positive or pT3 or pT4 bladder cancer patients

We also conducted a multivariable Cox regression analysis, selecting age, lymph node positivity (LND), and PT staging as predictive factors for OS. Early studies suggested that in patients undergoing RC for bladder cancer, the perioperative mortality and postoperative complication rates significantly increase with advanced age^27,28^. However, with advancements in medical technology, this difference has gradually diminished. Currently, there is no significant difference in perioperative mortality and early postoperative complication rates between elderly and younger patients^21,29^. From a prognostic perspective, age is an important prognostic factor for patients undergoing RC^29^. As age increases, the risk of recurrence rises while tumor-specific survival decreases. However, most literature suggests that age is not an independent prognostic factor and is correlated with patient’s pathological T stage and tumor grade. Previous reports^30^ have shown that for nonmuscle-invasive urothelial carcinoma, there is no significant difference in prognosis between elderly and younger patients. In our study, incorporating conclusions from 10 studies (HR: 1.63 (1.37–1.94), *P*=0.054), similar to other research findings, age is not a predictive factor for OS prognosis in bladder cancer patients.

Clinical TNM staging is an important indicator for clinical decision-making and prognostic evaluation, and different stages of bladder cancer patients can receive different adjuvant treatments, which can also ensure thorough clearance of pelvic lymph nodes with tumor metastasis. In this study, we included an analysis of the impact of staging >T3 on patients’ OS rate based on eight literature sources (HR: 1.02 (1.0–1.03), *P*=0.008).

LND, calculated as the ratio of positive lymph nodes to the total number of resected lymph nodes, has been introduced as a new method for stratifying PN+ patients^31^. It has been reported that LND is a useful prognostic tool as it reflects the disease burden of nodal disease during RC and the rigor of nodal clearance^32,33^. Additionally, multiple studies have reported that LND is superior to TNM lymph node staging in predicting post-RC survival rates^33–35^. Consistent with previous research, in this study (HR: 1.60 (1.31–1.95) *P*=0.000), LND emerged as an independent predictor of overall mortality, and pathological N stage could not predict prognosis.

### Limitations

This study has several limitations that need to be considered. Firstly, we did not have a standardized postoperative chemotherapy regimen for patients. Two articles used Carboplatin-based chemotherapy regimens, nine articles used Cisplatin-based regimens, and ten articles did not specify the chemotherapy regimen used. This variation may contribute to the heterogeneity in outcome measures. The latest guidelines from the European Association of Urology in 2023 strongly recommend providing lymph node-positive patients with platinum-based adjuvant combination chemotherapy^20^. Therefore, we did not compare the advantages and disadvantages of the two in terms of treatment. Secondly, due to the different definitions of the number of lymph node metastases in current studies, it was not possible to conduct a meta-analysis, Third, among the 21 articles we included, only one was a RCT, with the rest comprising six prospective studies and fourteen retrospective studies. The predominance of retrospective studies, which rely on existing data, could introduce selection bias, information bias, and recall bias, potentially leading to an overestimation of our results. Fourth, the time points for the observed outcome measures in the included studies were not completely consistent, which could introduce heterogeneity into the results. Necessitating more central randomized clinical trials to confirm and validate.

Currently, PN+ patients are often combined with pT3/4 patients, and there has been no meta-analysis specifically analyzing PN+ bladder cancer patients so far^21^. Studies have shown a trend towards greater benefit from postoperative chemotherapy in PN+ patients. Therefore, this study conducted a separate analysis of lymph node-positive bladder cancer patients, suggesting that compared to pT3/4 patients, lymph node-positive patients benefit more from postoperative AC. We also found that LND is an independent predictor of overall mortality, and its predictive effect is superior to TNM lymph node staging^23^.

## Conclusion

Our study results indicate that postoperative AC can improve OS, tumor-specific survival, and RFS in patients with pT3, pT4, and lymph node-positive bladder cancer. Additionally, lymph node-positive patients benefit more from postoperative AC treatment. Therefore, compared to pT3/4 patients, lymph node-positive patients should be more actively considered for postoperative AC in clinical practice to obtain greater benefits. Currently, both clinical experience and the latest guidelines strongly recommend the use of platinum-based postoperative AC regimens. It was further demonstrated that cisplatin-based chemotherapy regimens are more beneficial than other regimens for patients with locally advanced bladder cancer. However, these results still need to be confirmed by further large-scale, prospective, multicenter, long-term follow-up RCTs.

## Ethical approval

Not applicable.

## Consent

Consent for publication: Not applicable.

Consent to participate: Not applicable.

## Source of funding

Not applicable.

## Author contribution

C.X.-C.: study concept or design; C.X.-C., C.J.-W, H.T.-H., and H.Y.-L.: data collection; Y.L. and Z.W.: data analysis or interpretation; C.X.-C.: writing the paper. All authors contributed to the study’s conception and design. All authors commented on previous versions of the manuscript. All authors read and approved the final manuscript.

## Conflicts of interest disclosure

The authors declare that they have no conflict of interest.

## Research registration unique identifying number (UIN)

CRD42024505526.

## Guarantor

Xue‑song Yang.

## Data availability statement

The original contributions presented in the study are included in the article material, further inquiries can be directed to the corresponding author/s.

## Provenance and peer review

Not commissioned, externally peer-reviewed.
